# Efficient and stable perovskite solar cells prepared in ambient air irrespective of the humidity

**DOI:** 10.1038/ncomms11105

**Published:** 2016-04-01

**Authors:** Qidong Tai, Peng You, Hongqian Sang, Zhike Liu, Chenglong Hu, Helen L. W. Chan, Feng Yan

**Affiliations:** 1 Department of Applied Physics, The Hong Kong Polytechnic University, Hung Hom, 999077 Kowloon, Hong Kong; 2 Institute for Interdisciplinary Research and Key Laboratory of Optoelectronic Chemical Materials and Devices of Ministry of Education, Jianghan University, 430056 Wuhan, China

## Abstract

Poor stability of organic–inorganic halide perovskite materials in humid condition has hindered the success of perovskite solar cells in real applications since controlled atmosphere is required for device fabrication and operation, and there is a lack of effective solutions to this problem until now. Here we report the use of lead (II) thiocyanate (Pb(SCN)_2_) precursor in preparing perovskite solar cells in ambient air. High-quality CH_3_NH_3_PbI_3−*x*_(SCN)_*x*_ perovskite films can be readily prepared even when the relative humidity exceeds 70%. Under optimized processing conditions, we obtain devices with an average power conversion efficiency of 13.49% and the maximum efficiency over 15%. In comparison with typical CH_3_NH_3_PbI_3_-based devices, these solar cells without encapsulation show greatly improved stability in humid air, which is attributed to the incorporation of thiocyanate ions in the crystal lattice. The findings pave a way for realizing efficient and stable perovskite solar cells in ambient atmosphere.

## Abstract (web summary)

Poor stability of perovskite solar cells in humid conditions has hindered device fabrication and operation. Here, Tai *et al*. report the use of a lead thiocyanate precursor in preparing perovskite solar cells and without encapsulation showed better stability in air than typical perovskite solar cells.

## Graphical abstract

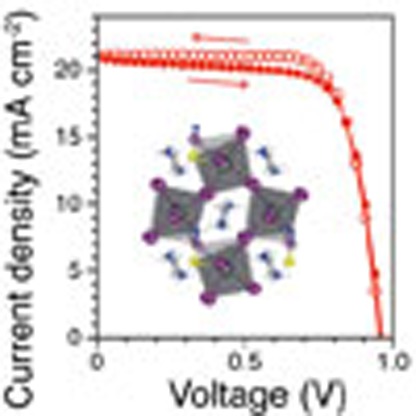

Organolead halide (CH_3_NH_3_PbX_3_, X=Cl, Br or I) perovskite solar cells have stepped into the spotlight of solar cell community for an unprecedented increase of their efficiencies from 3.8% to over 20% in <5 years^1^,^2^,^3^,^4^,^5^,^6^,^7^,^8^,^9^,^10^,^11^. CH_3_NH_3_PbX_3_ can be synthesized by the reaction of PbX_2_ and CH_3_NH_3_X via one- or two-step solution processing methods^3^,^4^, vapour deposition methods^5^ or vapour-assisted solution methods^12^. Besides the unique features of strong light absorption, ambipolar charge transport, long carrier lifetime and solution processability, CH_3_NH_3_PbX_3_ perovskites have distinguished themselves from other solar cell materials for being compatible with a wide range of device architectures^11^,^13^,^14^.

Despite the success in obtaining excellent photovoltaic performance, the instability of CH_3_NH_3_PbX_3_ to water and ambient moisture is still an open problem^15^. In most cases, perovskite films have to be processed in inert atmosphere and bare devices cannot survive long in air, which hamper the real production and applications of the perovskite solar cells. So far, effective solutions to this issue still lack although some related studies have been carried out^16^,^17^,^18^. For example, water-resistive coating can be used to improve the stability of perovskite solar cells; however, the intrinsic vulnerability of perovskites to moisture remains unchanged^19^. Better moisture stability was observed for a two-dimensional, layered perovskite formed by incorporating phenylethylammonium (C_6_H_5_(CH_2_)_2_NH_3_^+^) into CH_3_NH_3_PbI_3_ matrix. Such a perovskite film could be fairly fabricated in ambient condition, while its photovoltaic performance was less satisfactory^18^. Similar observations were also reported for mixed halide perovskites CH_3_NH_3_PbI_3-*x*_Br_*x*_, when 10–15 mol% I^−^ was substituted by Br^−^. The enhanced stability was attributed to the stronger interaction between Br^−^ and CH_3_NH_3_^+^ (refs 6, 16). These studies suggest that ion doping might be a possible way to improve the inherent stability of perovskite films.

It is also perceived that both the photovoltaic performance and stability of perovskite films are closely related to the film quality that is governed by the methods and materials used. To date, PbI_2_ and PbCl_2_ have been ubiquitously used to prepare the state-of-art CH_3_NH_3_PbI_3_ perovskite films, and in the latter case, the perovskite is often referred as CH_3_NH_3_PbI_3−*x*_Cl_*x*_ although the existence of Cl in the final film is still under debate^20^,^21^. Recent studies showed that non-halide lead precursors could also be used to prepare high-quality perovskite films^22^,^23^,^24^,^25^. Although no superior device performance has been obtained yet, such precursors would lead to versatile approaches for the preparation of novel perovskite films with different optoelectronic properties and air stability.

Here we report a type of efficient and stable perovskite solar cells by using lead (II) thiocyanate (Pb(SCN)_2_) precursor. The perovskite layer was formed on mesoporous TiO_2_ (mp-TiO_2_) scaffold in ambient air by a two-step sequential deposition method, as shown in Fig. 1a. Fine perovskite films can be readily prepared in our lab even when the average relative humidity (RH) exceeds 70%, suggesting excellent moisture stability of the perovskite. Upon optimization, the devices demonstrated average power conversion efficiencies (PCEs) over 13%, together with the currently maximum value higher than 15%. More importantly, these devices without encapsulation showed much better stability in ambient air than typical perovskite solar cells prepared from PbI_2_ precursor.

## Results

### Preparation of perovskite solar cells

**Figure 1:**
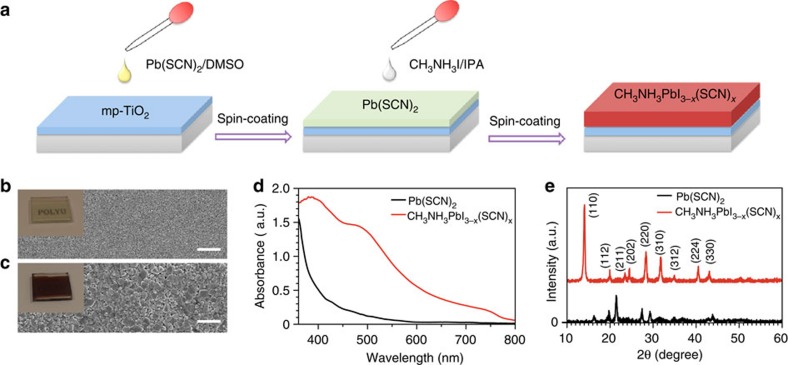
**Fabrication and characterization of CH**_**3**_**NH**_**3**_**PbI**_**3-*****x***_**(SCN)**_***x***_ **perovskite film.** (**a**) Schematic illustration of the method used for preparing perovskite films. Digital and SEM images of (**b**) Pb(SCN)_2_ film and (**c**) the resulting perovskite film (CH_3_NH_3_PbI_3-*x*_(SCN)_*x*_). (**d**) Absorption spectra and (**e**) XRD patterns of Pb(SCN)_2_ film and the resulting perovskite film (CH_3_NH_3_PbI_3-*x*_(SCN)_*x*_). Scale bars=2 μm (**b**,**c**).

As shown in Fig. 1a, dimethylsulfoxide (DMSO) was used as a solvent to prepare Pb(SCN)_2_ films, and highly dense and uniform film morphology was obtained (Fig. 1b), which should be beneficial for obtaining high-quality perovskite films. In contrast, a quite poor film morphology was found in the Pb(SCN)_2_ film derived from the commonly used solvent *N*,*N*′-dimethylformamide (Supplementary Fig. 1). After reacting with CH_3_NH_3_I, both the colour and morphology of Pb(SCN)_2_ films changed strikingly (Fig. 1c). The resulting films exhibited quite similar absorption spectrum and X-ray diffraction pattern to the state-of-art tetragonal CH_3_NH_3_PbI_3_ perovskite (Fig. 1d,e; ref. 4). It is notable that both X-ray diffraction and Raman spectroscopy (Supplementary Fig. 2) do not show any peak of Pb(SCN)_2_ after the reaction, suggesting a complete conversion of Pb(SCN)_2_ into perovskite in the film. On the other hand, S was clearly found in the final perovskite films of different batches under X-ray photoelectron spectroscopy (XPS; Supplementary Fig. 3). The feature characteristic of S 2*p* core electrons was observed, and the concentration of S was estimated to be 2.7–8.4 mol% with respect to I. We speculate that the observed S atoms are from SCN^−^ incorporated into CH_3_NH_3_PbI_3_ perovskite, although the concentration is rather low. Considering that SCN^−^ is known as a pseudohalide group with its ionic radius (0.215–0.22 nm) very close to that of I^−^ (0.22 nm; ref. 26), it is possible for some of SCN^−^ to substitute I^−^ in the perovskite lattice. This speculation is also supported by recent studies in which the incorporation of BF_4_^−^ (with a similar ionic radius of 0.218 nm) into CH_3_NH_3_PbI_3_ perovskite was theoretically and experimentally confirmed^27^,^28^. Therefore, the perovskite prepared in this work will be noted as CH_3_NH_3_PbI_3-*x*_(SCN)_*x*_ hereafter. When the CH_3_NH_3_PbI_3−*x*_(SCN)_*x*_ film was left to degrade in moisture, the final product was mainly identified as PbI_2_ (Supplementary Fig. 4), indicating that Pb(SCN)_2_ follows similar reaction mechanism with PbCl_2_ and lead acetate (PbAc_2_), and most of the SCN^−^ ions have evaporated during the annealing of perovskite film^20^,^21^,^25^.

**Figure 2:**
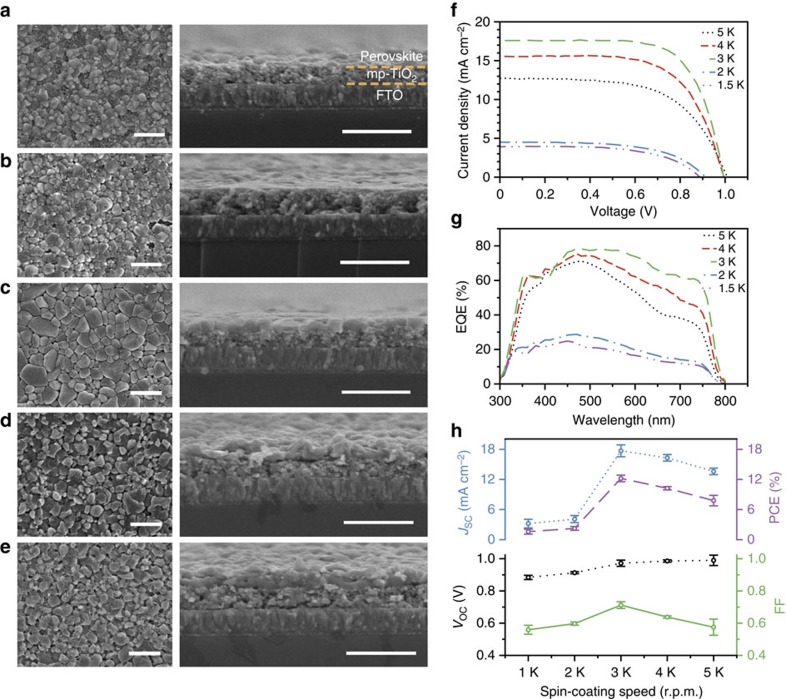
**Morphology and performance of CH**_**3**_**NH**_**3**_**PbI**_**3**__**-*****x***_**(SCN)**_***x***_ **perovskite.** SEM top view and cross-sectional view images of CH_3_NH_3_PbI_3-*x*_(SCN)_*x*_ films derived from Pb(SCN)_2_ deposited on mp-TiO_2_ films at the spin-coating speeds of (**a**) 5,000 r.p.m., (**b**) 4,000 r.p.m., (**c**) 3,000 r.p.m., (**d**) 2,000 r.p.m. and (**e**) 1,500 r.p.m., respectively. Scale bars=1 μm (**a**–**e**). The representative (**f**) *J–V* curves, (**g**) EQEs and (**h**) statistical photovoltaic parameters (*J*_SC_, PCE, *V*_OC_ and FF) of CH_3_NH_3_PbI_3-*x*_(SCN)_*x*_ perovskite solar cells prepared at different spin-coating conditions. Error bars represent s.d. calculated from five devices prepared at the same conditions.

Figure 2a–e shows the scanning electron microscope (SEM) images of the perovskite films prepared at different conditions. The spin-coating speed used for the deposition of Pb(SCN)_2_ has a significant influence on the morphology and thickness of the final perovskite films. Flat polygonal-grained morphologies are observed for perovskite films prepared at the spin-coating speed higher than 3,000 r.p.m. and the grain size increases with decreasing speed. However, according to the cross-sectional views, incomplete coverage on mp-TiO_2_ is found for the perovskite films prepared at the spin-coating speeds above 4,000 r.p.m., and 100% surface coverage (upper layer thickness: ∼140 nm) is only achievable at the intermediate speed of 3,000 r.p.m. On the other hand, very rough structures are observed for perovskite films prepared at 2,000 and 1,500 r.p.m. with the upper layer thicknesses of ∼240 and ∼260 nm, respectively. Such a rough surface morphology is mainly due to the high viscosity of DMSO that cannot be spread well at low spin-coating speeds.

Then perovskite solar cells were fabricated with a device configuration of fluorine doped tin oxide (FTO)/compact TiO_2_/mp-TiO_2_/perovskite/2,2′,7,7′-Tetrakis(*N,N*-di-*p*-methoxyphenyl amine)-9,9′ -spirobifluorene (spiro- MeOTAD)/Au, as shown in Supplementary Fig. 5, and their photovoltaic performance was characterized systematically. The representative current density–voltage (*J-V*) characteristics, corresponding external quantum efficiencies (EQEs) and photovoltaic parameters, are presented in Fig. 2f,g. Perovskites prepared at the speeds of 5,000 r.p.m. and 4,000 r.p.m. give average open-circuit voltages (*V*_OC_) of 0.99±0.03 and 0.98±0.01 V, short circuit current densities (*J*_SC_) of 13.6±0.6 and 16.3±0.7 mA cm^−2^, fill factors (FFs) of 0.575±0.05 and 0.638±0.007, and PCEs of 7.79±1.05% and 10.24±0.32%, respectively. Improved *J*_SC_ of 17.7±1.1 mA cm^−2^, FF of 0.712±0.021 and PCE of 12.20±0.66% are obtained in the case of 3,000 r.p.m. while the change of *V*_OC_ is negligible. The increase of *J*_SC_ is attributed to the improved EQE values in the long wavelength region (500–800 nm) for the increase of the film thickness. Improved film coverage at the intermediate spin-coating speed accounts for the increase of FF as the interface carrier recombination is reduced. In contrast, striking drop of the PCE to ∼2% is observed for perovskites prepared at 2,000 and 1,500 r.p.m., which is mainly caused by the reduction of *J*_sc_. This phenomenon can be understood by the following reasons^29^: (1) the perovskite layer is too thick to have efficient charge separation; (2) the poor contact between crystals in the perovskite film may hamper the charge transport process; and (3) the rough surface of the perovskite film may cause an incomplete coverage of the hole transport layer, resulting in direct carrier recombination at the perovskite/Au interface.

### Performance of the CH_3_NH_3_PbI_3-*x*_(SCN)_*x*_ perovskite solar cells

**Figure 3:**
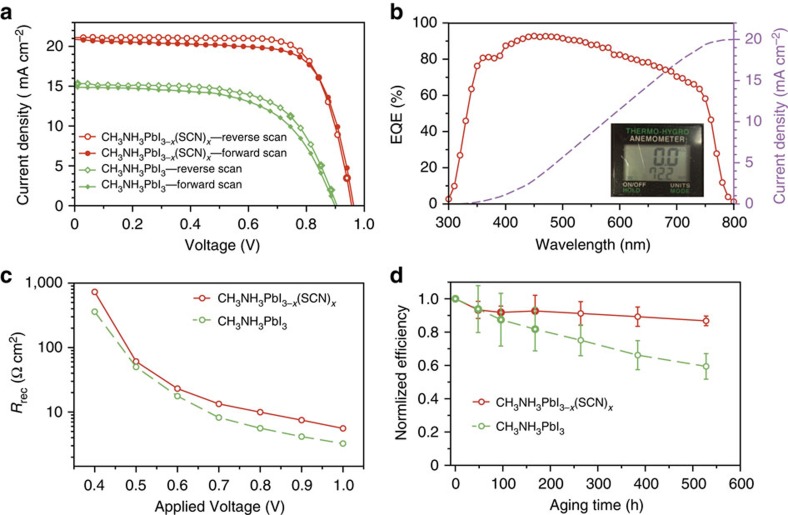
**Characteristics of CH**_**3**_**NH**_**3**_**PbI**_**3-*****x***_**(SCN)**_***x***_ **perovskite solar cells and control devices.** (**a**) *J–V* curves of CH_3_NH_3_PbI_3-*x*_(SCN)_*x*_ (Pb(SCN)_2_-derived) and CH_3_NH_3_PbI_3_ (PbI_2_-derived)-based solar cells prepared in ambient air. (**b**) Corresponding EQE of the CH_3_NH_3_PbI_3-*x*_(SCN)_*x*_-based device. The inset photo shows the RH of the ambient air of 72.2%. (**c**) The representative recombination resistances (*R*_rec_) determined from IS under different applied bias voltages. (**d**) Evolution of the PCEs of CH_3_NH_3_PbI_3-*x*_(SCN)_*x*_- and CH_3_NH_3_PbI_3_-based solar cells upon ageing in air without encapsulation. Error bars represent s.d. calculated from five devices prepared at the same conditions.

By further optimizing the fabrication conditions of mp-TiO_2_ layer, higher PCEs can be obtained from devices fabricated in open air regardless of the ambient moisture. As shown in Fig. 3a,b, a device shows the PCE of 15.12% at reverse scan and 14.52% at forward scan, despite the fact that the RH of the ambient air is above 70%. The average PCE of 20 devices prepared at the same conditions is 13.49±1.01% (Supplementary Fig. 6), suggesting an excellent moisture stability of CH_3_NH_3_PbI_3-*x*_(SCN)_*x*_ perovskite. We also noticed that the stabilized PCE of a CH_3_NH_3_PbI_3-*x*_(SCN)_*x*_ solar cell characterized in humid air is very close to that obtained from its *J–V* curves (Supplementary Fig. 7).

To better illuminate the moisture effect, control CH_3_NH_3_PbI_3_ solar cells were fabricated in air under the same conditions except using PbI_2_ precursor with DMSO solvent. As shown in Fig. 3a, the control device shows the PCEs of 8.78% and 8.02% in the reverse scan and forward scan, respectively. The detailed photovoltaic parameters of the two devices are summarized in Supplementary Table 1. One possible reason for the low efficiency of the control device is the poor morphology of CH_3_NH_3_PbI_3_ film caused by the ambient high RH, which is rough and full of pinholes. SEM images show that the uncovered regions in some places are larger than 1 μm scale (Supplementary Fig. 8). Such a poor morphology will cause severe recombination for the direct contact between hole-transporting material (HTM) and TiO_2_. In contrast, the CH_3_NH_3_PbI_3_ solar cells prepared in controlled atmosphere (N_2_-filled glovebox) with the same PbI_2_ precursor show higher efficiencies of ∼12% due to the much improved film morphology (Supplementary Fig. 9). The above results demonstrate that the preparation of perovskite solar cells is more moisture resistive through Pb(SCN)_2_ route than through PbI_2_ route.

To further explore the origin of the different performance of CH_3_NH_3_PbI_3-*x*_(SCN)_*x*_- and CH_3_NH_3_PbI_3_-based devices, impedance spectroscopy (IS) measurements were performed and the corresponding IS spectra are presented in Supplementary Fig. 10. As reported in the literature^30^,^31^,^32^,^33^, the IS response of a perovskite solar cell is composed of three features (arcs in Nyquist plot). The first arc in high-frequency region is possibly related to the charge transport in the TiO_2_ layer, HTM and/or at HTM/Au interface^30^,^31^,^32^,^33^,^34^; this feature may become ambiguous if the charge transport resistance (*R*_HTM_) is low. The second arc in the intermediate-frequency region is associated with the charge transport-recombination process in the active film, from which the recombination resistance (*R*_rec_) can be obtained and this feature is typically dominant in the IS response; The third arc in the low-frequency region that usually appears under a high applied voltage is correlated to slow dynamic processes in the perovskite film. Here the second arcs in the intermediate-frequency region can be clearly observed in all of the devices, which reflect the carrier recombination processes.

Figure 3c shows the *R*_rec_ values of the two devices under different applied voltages. Both devices show a similar decrease of *R*_rec_ with increasing bias voltage due to increased carrier densities^31^,^35^, and a higher *R*_rec_ value is observed in the CH_3_NH_3_PbI_3-x_(SCN)_x_ solar cell at any bias voltage in comparison with the control device, thereby indicating a slower recombination rate in the former, which is favourable for device performance. Thus, the observation is consistent with the better performance of the CH_3_NH_3_PbI_3-*x*_(SCN)_*x*_ solar cell than the control device. The reduced recombination in the CH_3_NH_3_PbI_3-*x*_(SCN)_*x*_ solar cell can be attributed to two possible reasons. One is the pinhole-free morphology of the CH_3_NH_3_PbI_3-*x*_(SCN)_*x*_ film, which can avoid direct carrier recombination at HTM/TiO_2_ interface. The other is the lower density of trap states in CH_3_NH_3_PbI_3-*x*_(SCN)_*x*_ than in CH_3_NH_3_PbI_3_ film, as evidenced in the following experiments.

The carrier recombination processes in the perovskite films were characterized by time-resolved photoluminescence measurements (Supplementary Fig. 11). The lifetimes related to the recombination of free carriers in CH_3_NH_3_PbI_3-*x*_(SCN)_*x*_ and CH_3_NH_3_PbI_3_ films are extracted to be 260.35 and 30.5 ns, respectively. Obviously, the 8.5-times-longer carrier lifetime in the former indicates lower trap density in the CH_3_NH_3_PbI_3-*x*_(SCN)_*x*_ film. In addition, it has been theoretically and experimentally proved that lower density of trap states can be found in perovskite film grown from iodide-free precursor than that grown from iodide-containing precursor^22^. Hence, we can conclude that the CH_3_NH_3_PbI_3-*x*_(SCN)_*x*_ solar cells have longer carrier lifetime and lower trap density than the control devices, which is a major reason for their better performance.

### Stability of perovskite solar cells in humid air

Since excellent moisture resistance has been observed in CH_3_NH_3_PbI_3-*x*_(SCN)_*x*_ solar cells during the fabrication process, it is not surprising to find a good long-term ambient stability of them. As shown in Fig. 3d, CH_3_NH_3_PbI_3-*x*_(SCN)_*x*_ solar cells without encapsulation maintain 86.7% of the initial average PCE after being stored in open air with the average RH level above 70% for over 500 h. In comparison, the CH_3_NH_3_PbI_3_ control devices lose nearly 40% of the initial average PCE (see the evolution of *V*_OC_, *J*_SC_, and FF in Supplementary Fig.12).

It has been reported that the morphology of a CH_3_NH_3_PbI_3_ perovskite film may influence its stability in air^17^. Since the CH_3_NH_3_PbI_3-*x*_(SCN)_*x*_ film shows more uniform and compact surface morphology than the CH_3_NH_3_PbI_3_ film prepared in air, the better morphology of the former is also a possible reason for the improved ambient stability of the devices. To clarify this issue, CH_3_NH_3_PbI_3_ solar cells prepared in controlled atmosphere with the efficiency of ∼15% were tested for hundreds of hours in ambient air (Supplementary Fig. 13). In these control devices, the morphology of the CH_3_NH_3_PbI_3_ film is similar to that of the CH_3_NH_3_PbI_3-*x*_(SCN)_*x*_ film shown in Fig. 2c. However, the PCEs of the devices decreased rapidly with time, indicating that the morphology of the films plays a minor role on the stability of the devices in air with high RH. These results clearly demonstrate that the intrinsic stability of the perovskite material is the dominating factor for the device stability in our experiments.

**Figure 4:**
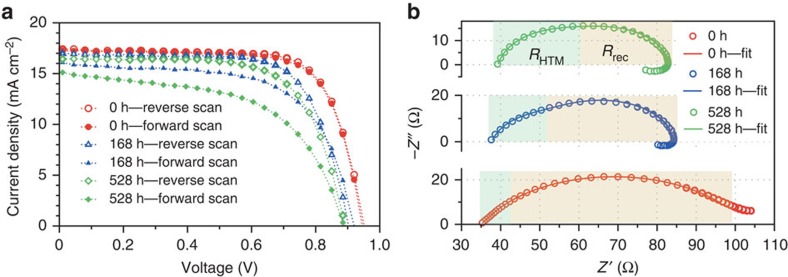
**Ageing tests of a CH**_**3**_**NH**_**3**_**PbI**_**3-x**_**(SCN)**_**x**_ **perovskite solar cell.** (**a**) *J–V* curves and (**b**) IS spectra (1 V bias voltage in dark) of a CH_3_NH_3_PbI_3-*x*_(SCN)_*x*_ solar cell with characteristic ageing time of 0, 168 and 528 h. The high- and intermediate-frequency responses in the IS are fitted with the equivalent circuit shown in Supplementary Fig. 5, and the corresponding charge transfer resistance in the hole transport material (*R*_HTM_) and the recombination resistance (*R*_rec_) of the device are highlighted with different colours. The width of each region represents the value of the corresponding resistance.

Although little *J–V* hysteresis can be found in a fresh CH_3_NH_3_PbI_3-*x*_(SCN)_*x*_ solar cell, it becomes obvious upon ageing of the device in ambient air. The similar change of *J*–*V* hysteresis has been observed in the ageing tests of CH_3_NH_3_PbI_3_ solar cells in either this work or other reports^36^. Based on IS analysis, we are able to get an insight into the origin of such an ageing-dependent *J–V* hysteresis as well as the degradation mechanism of perovskite solar cells. Figure 4a displays the *J–V* curves of a CH_3_NH_3_PbI_3−*x*_(SCN)_*x*_ solar cell measured at three representative ageing times, that is, initial stage (0 h), middle stage (168 h), when the hysteresis starts to be obvious, and final stage (528 h). Figure 4b shows the corresponding IS spectra measured in dark with 1 V bias voltage, and the derived *R*_HTM_ and *R*_rec_ values are presented in Supplementary Table 2. Since the *V*_OC_ of the aged device decreased to ∼0.9 V, the change of *R*_HTM_ and *R*_rec_ values were also measured with a 0.9-V applied voltage (Supplementary Fig. 14), and the trend is similar. Clearly, *R*_HTM_ increases gradually with ageing time, whereas *R*_rec_ goes in the opposite direction. As mentioned above, the former indicates an increased charge transport resistance in TiO_2_ or Spiro-MeOTAD HTM layer or at the HTM/Au interface, whereas the latter means an increased recombination rate in the device. Both issues may lead to the degradation of the device performance.

Since the TiO_2_ layer and Au electrode are very stable in air, the increased *R*_HTM_ is presumably caused by the hygroscopic nature of Li-bis(trifluoromethanesulfonyl)-imide (Li-TFSI), which is typically used as a p-type dopant to increase the conductivity of Spiro-MeOTAD^37^,^38^. Meanwhile, the Spiro-MeOTAD oxidation might also account for the increase of *R*_HTM_ (ref. 39). To clarify this point, a control experiment was conducted, in which the perovskite solar cells were kept in dry air and this would allow us to focus on the influence of air exposure on *R*_HTM_ without considering the moisture effect. We did not observe much change of the *R*_HTM_ values, even when the devices were exposed to dry air for 168 h (Supplementary Fig. 15). In contrast, the *R*_HTM_ values almost doubled in humid air (Fig. 4b), indicating that the Spiro-MeOTAD oxidation is not a reason for the increased *R*_HTM_.

The reduction of *R*_rec_ upon ambient air exposure is related to the moisture-induced defects in the perovskite, which is probably caused or accelerated by the presence of Li-TFSI^19^,^37^,^38^, and these defects account partially for the ageing-induced *J–V* hysteresis^36^,^40^. Besides, negative capacitance is observed in the aged device in the low-frequency region, which can enhance the hysteresis since it is closely related to a slow relaxation process in the device^32^. The physical origin of such a negative capacitance is still unclear, which may be related to the interfacial ion reorganization or dielectric relaxation in a perovskite film^30^,^32^,^33^. In our case, either origin should be associated with the air exposure that can induce more traps in the active layers.

### Theoretical calculation of CH_3_NH_3_PbI_3-*x*_(SCN)_*x*_ perovskite

**Figure 5:**
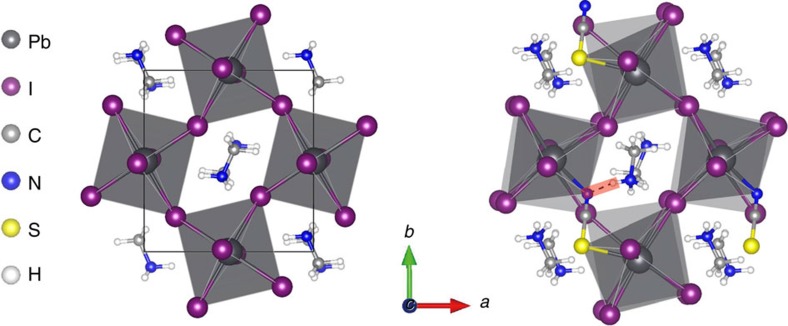
Theoretical calculation of perovskite materials. Calculated crystal structures of CH_3_NH_3_PbI_3_ (left) and CH_3_NH_3_PbI_3−*x*_(SCN)_*x*_ based on a chemical formula of (CH_3_NH_3_)_4_Pb_4_I_11_SCN(right). The hydrogen bond between SCN^−^ and CH_3_NH_3_^+^ (dash line) is highlighted with red colour.

For a better understanding of the structural and chemical properties of CH_3_NH_3_PbI_3-*x*_(SCN)_*x*_, density functional theory calculations were conducted (Fig. 5). Here *x*=0.25, corresponding to the ratio of SCN^−^ to I^−^ numbers in the lattice of ∼9%, which is close to the maximum molar ratio (8.4%) of SCN^−^ obtained by the XPS measurement. Our calculations show that the incorporation of SCN^−^ group into the perovskite lattice is thermodynamically stable. In case of CH_3_NH_3_PbI_3_, orthorhombic structure is energetically favoured and the CH_3_NH_3_^+^ aligns in parallel along *b* axis (the orthorhombic phase will change into tetragonal at *T*>162 K due to the free rotation of CH_3_NH_3_^+^ and the influence of temperature is not taken into account in our calculations^41^). The stabilized CH_3_NH_3_PbI_3-*x*_(SCN)_*x*_ shows a pseudo-orthorhombic structure, as the SCN^−^ group tends to align along the same direction as CH_3_NH_3_^+^, resulting in a slight tilt of the crystal lattice (the lattice tilt could be greatly alleviated at room temperature owing to the small amount of SCN^−^ and the free rotation of CH_3_NH_3_^+^). The band structure of CH_3_NH_3_PbI_3-*x*_(SCN)_*x*_ was calculated (Supplementary Fig. 16), which is similar to that of CH_3_NH_3_PbI_3_. Fortunately, no electronic levels (traps) are introduced in the gap, which is critical to carrier recombination process in the perovskite material. This theoretical result is consistent with the observed long carrier lifetime of the material in the photoluminescence measurement. More importantly, our calculations indicate strong ionic interactions between SCN^−^ and adjacent Pb atoms, and hydrogen bonds can be formed between SCN^−^ and CH_3_NH_3_^+^, which should contribute to improved chemical stability of perovskites. This is very similar to the case for Br-doped perovskite that shows improved air stability for enhanced chemical bonding in perovskite lattice^6^,^16^.

## Discussion

According to the above study, the much better moisture resistance of CH_3_NH_3_PbI_3-*x*_(SCN)_*x*_ film can be mainly attributed to its good intrinsic stability of the perovskite material. In a very recent study, Jiang *et al*.^42^ reported an improved moisture stability of CH_3_NH_3_PbI_3_ perovskite by replacing two I^−^ with SCN^−^. Although the methodology, composition and performance of their perovskite solar cells are quite different from our work, the finding of the better moisture stability of SCN^−^ containing perovskite is shared.

In conclusion, we report the use of Pb(SCN)_2_ as a precursor for preparing perovskite solar cells in ambient air with RH higher than 70%, and a CH_3_NH_3_PbI_3-*x*_(SCN)_*x*_ formula is adopted for the resulting perovskite based on elemental analysis and theoretical calculations. The CH_3_NH_3_PbI_3-*x*_(SCN)_*x*_ perovskite exhibits less trap density and better intrinsic stability than conventional PbI_2_-derived CH_3_NH_3_PbI_3_ perovskite, and thus better and more stable device performance. The advantages of such perovskite solar cells are demonstrated by the PCEs up to 15% obtained without humidity control and highlighted by the little efficiency loss (<15%) upon long-term (>500 h) ageing in humid air without encapsulation. The slow decrease of the device efficiency with time is attributed to the increased charge transport resistance in Spiro-MeOTAD layer and the increased recombination rate in the perovskite. Ageing-induced *J–V* hysteresis is also observed during the stability test, which can be attributed to moisture-induced defects and slow dynamic processes (negative capacitance) in perovskite films. Further improvement of the device performance is expected by optimizing the morphology of mp-TiO_2_ films and the use of non-hygroscopic HTM. All of the findings will offer useful insights for obtaining efficient and stable perovskite solar cells at ambient conditions.

## Methods

### Preparation of TiO_2_ on electrodes

FTO glass was first patterned and cleaned, and then a 50–80-nm compact TiO_2_ layer was formed by spin-coating a 0.2-M titanium isopropoxide solution in ethanol with the addition of 0.02 M HCl at the spin-coating speed of 4,000 r.p.m., followed by sintering at 450 °C for 30 min. A ∼220-nm mp-TiO_2_ layer was then deposited by spin-coating TiO_2_ paste in ethanol at 3,000 r.p.m., which contains 4.5 wt% P25 (Degussa) and 5% (weight to TiO_2_) ethyl cellulose (22 cp). The paste was ground at 400 r.p.m. for 72 h before use. After that, the mp-TiO_2_ was sintered at 500 °C for 30 min, followed by 40-mM TiCl_4_ treatment at 70 °C for 30 min and sintering at 500 °C for another 30 min.

### Preparation of pervoskite solar cells

Pb(SCN)_2_ powder (Sigma-Aldrich) was dissolved in DMSO at 500 mg ml^−1^ and filtered with 0.45-μm nylon filter to get a clear solution. The solution was then spin-coated on mp-TiO_2_ scaffold and heated at 90 °C in air for 1 h to get Pb(SCN)_2_ film. Then CH_3_NH_3_I solution (10 mg ml^−1^ in isopropanol) was dropped on top of the Pb(SCN)_2_ film and kept for 20 s, followed by spin-coating at 3,000 r.p.m. for 20 s. This procedure was repeated three times to guarantee a complete conversion of Pb(SCN)_2_ into perovskite. The resulting perovskite film was rinsed with pure isopropanol and annealed at 80 °C in air for 20 min. The 2,2′,7,7′-Tetrakis(*N,N*-di-*p*-methoxyphenylamine)-9,9′ -spirobifluorene (spiro-MeOTAD) layer was prepared by spin-coating a chlorobenzene solution containing 80 mM spiro-MeOTAD, 64 mM tert-butylpyridine and 24 mM Li-TFSI (255 mg ml^−1^ in acetonitrile) at 4,000 r.p.m. for 60 s. Finally, 80-nm-thick Au electrode was deposited via thermal evaporation. The CH_3_NH_3_PbI_3_-based control devices were prepared by exactly the same method instead of using PbI_2_ precursor.

### Characterization

The optical and structural characterizations were performed on Shimadzu UV-2550 spectrophotometer and Rigaku SmartLab X-Ray diffractometer, respectively. The XPS was measured with VG Thermo Escalab 220i-XL. The film morphology was observed under JEOL JSM 6335F SEM. The *J–V* curves were measured under AM 1.5G-simulated illumination (Oriel 91160) with a power density of 100 mW cm^−2^, and the light intensity was calibrated with a standard reference cell (Oriel 91150 V). The scan rate of the measurements is ±0.03 V s^−1^. The EQE was measured with an EQE system containing a xenon lamp (Oriel 66902), a monochromator (Newport 66902), a Si detector (Oriel 76175_71580) and a dual-channel power meter (Newport 2931_C). The IS measurements of the devices were carried out in dark by using a HP 4294 impedance analyser in a frequency ranging from 40 Hz to 1 MHz with an oscillating voltage of 30 mV and applied DC bias voltages varying from 0 to 1.0 V.

### Calculation details

Density functional theory calculations were performed using VASP package^43^. The algorithm implemented in the code is based primarily on using plane-wave basis set and norm-conserving pseudopotentials. A plane wave with cutoff energy of 500 eV was sufficient to converge the total energy. The semi-local *d*-electrons of Pb were considered as valence electron in the calculation. The Perdew–Burke–Ernzerhof-generalized gradient approximation method^44^ was used as the exchange-correlation density functional. A gamma-centered *k*-point grid spacing of 0.2 Å^−1^ was used for reciprocal space integration in structure optimization and spacing of 0.1 Å^−1^ in electronic structure calculations. Geometry relaxation in most calculations was run until the forces on atoms that were allowed to relax were no >0.01 eV Å^−1^. Calculations were performed without taking into account spin–orbital coupling.

## Additional information

**How to cite this article:** Tai, Q. *et al*. Efficient and stable perovskite solar cells prepared in ambient air irrespective of the humidity. *Nat. Commun.* 7:11105 doi: 10.1038/ncomms11105 (2016).

## Supplementary Material

Supplementary Information Supplementary Figures 1-16 and Supplementary Tables 1 & 2.
