# Supplementary material for: Efficient and stable perovskite solar cells prepared in ambient air irrespective of the humidity
Source: Nat Commun. 2016 Apr 1;7:11105. doi: 10.1038/ncomms11105 (PMC4821988; doi:10.1038/ncomms11105)
Supplement: Supplementary Information — Supplementary Figures 1-16 and Supplementary Tables 1 & 2. [file ncomms11105-s1.pdf]

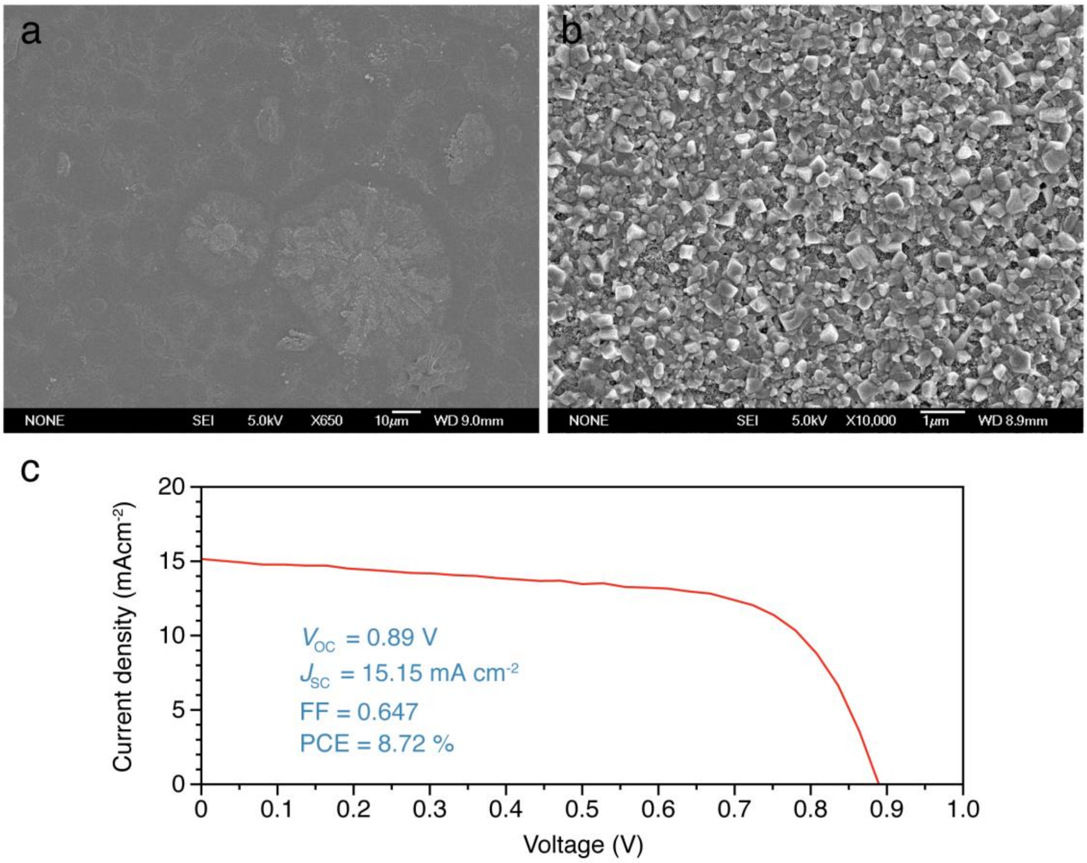

Supplementary Figure 1 | Morphology and photovoltaic performance characterizations. SEM images of (a) $Pb(SCN)_2$ film, (b) $CH_3NH_3PbI_{3-x}(SCN)_x$ film, and (c) *J*-*V* characteristic of $CH_3NH_3PbI_{3-x}(SCN)_x$ solar cell using DMF as solvent.

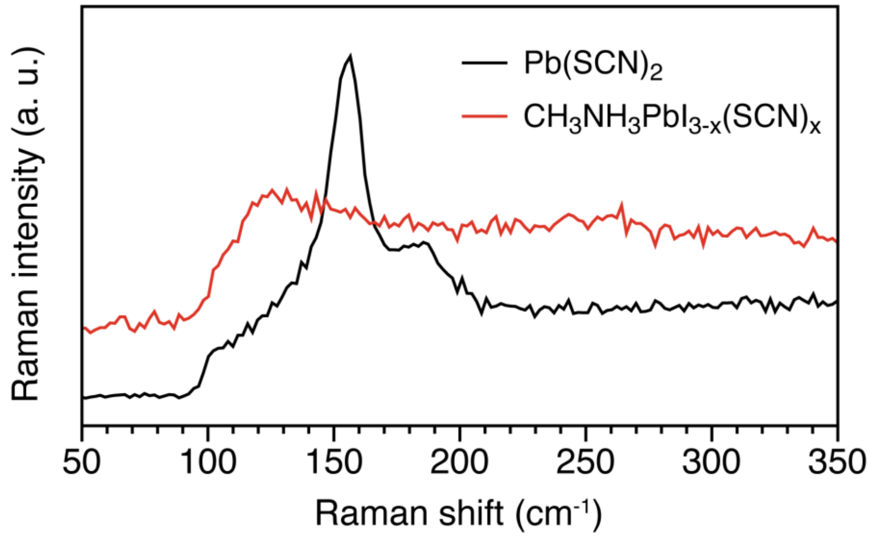

Supplementary Figure 2 | Raman Characterization. Raman spectra of $Pb(SCN)_2$ and $Pb(SCN)_2$-derived perovskite film.

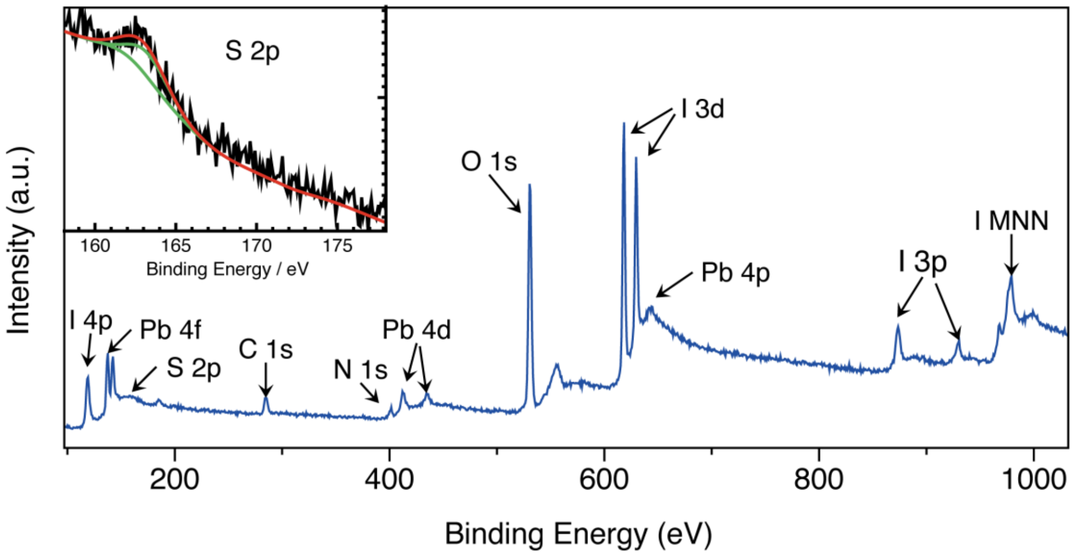

Supplementary Figure 3 | XPS characterization of the $Pb(SCN)_2$-derived perovskite film. The perovskite film was prepared on mesoporous TiO_2 substrate. The inset shows the experimental data (black) and fitted curve (red) for S 2p electrons.

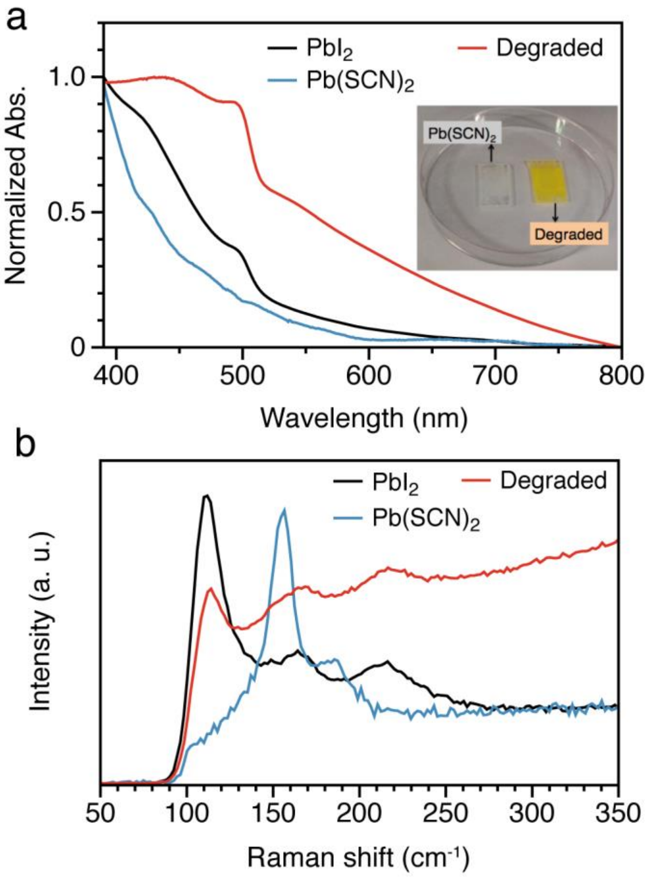

Supplementary Figure 4 | UV-Vis and Raman chararcterization. (a) Absorbance, and (b) Raman spectra of PbI_2, $Pb(SCN)_2$, and degraded $CH_3NH_3PbI_{3-x}(SCN)_x$ films.

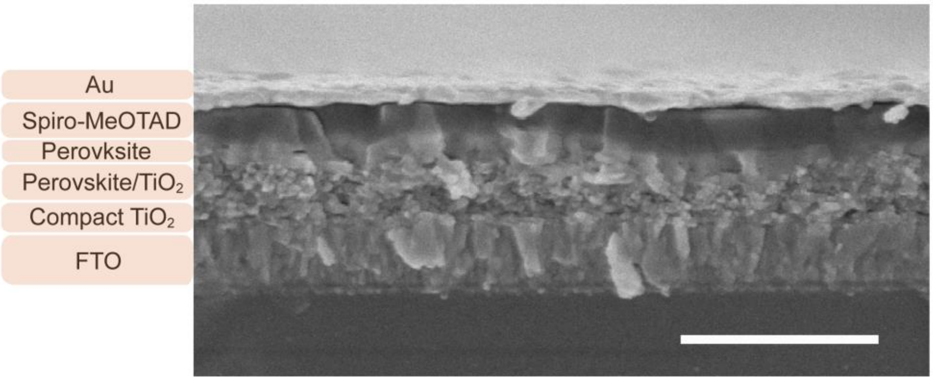

Supplementary Figure 5 | Solar cell morphology. SEM cross-section image (right) and the schematic (left) of a typical perovskite solar cell fabricated in this work. The scale bar is 1μm.

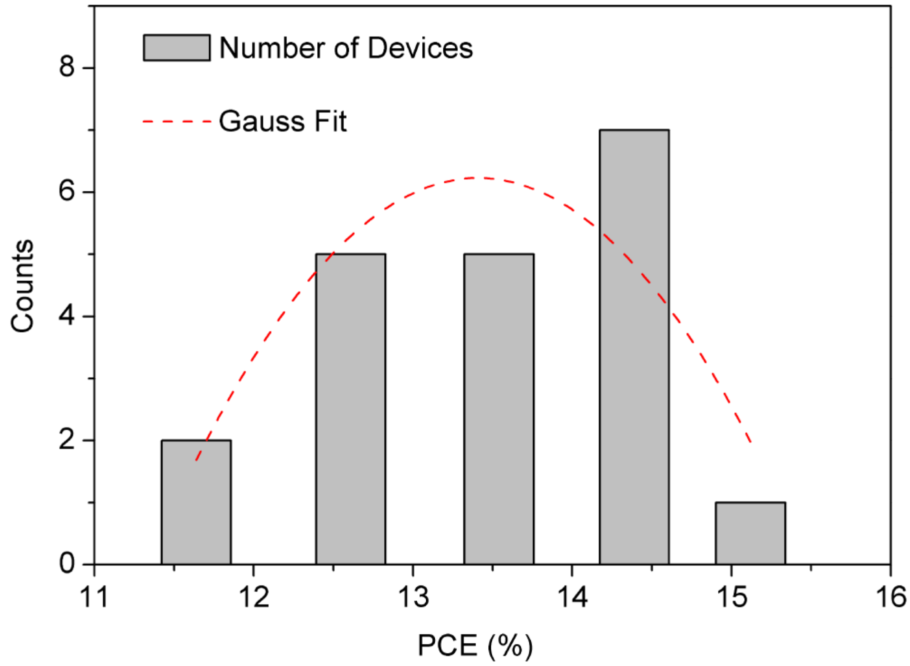

Supplementary Figure 6 | Histogram of solar cell efficiencies. The number of devices with the efficiencies lying in different regions between neighboring integer percentages for the same batch of 20 devices. The dash line represents the Gauss fit of the device distribution.

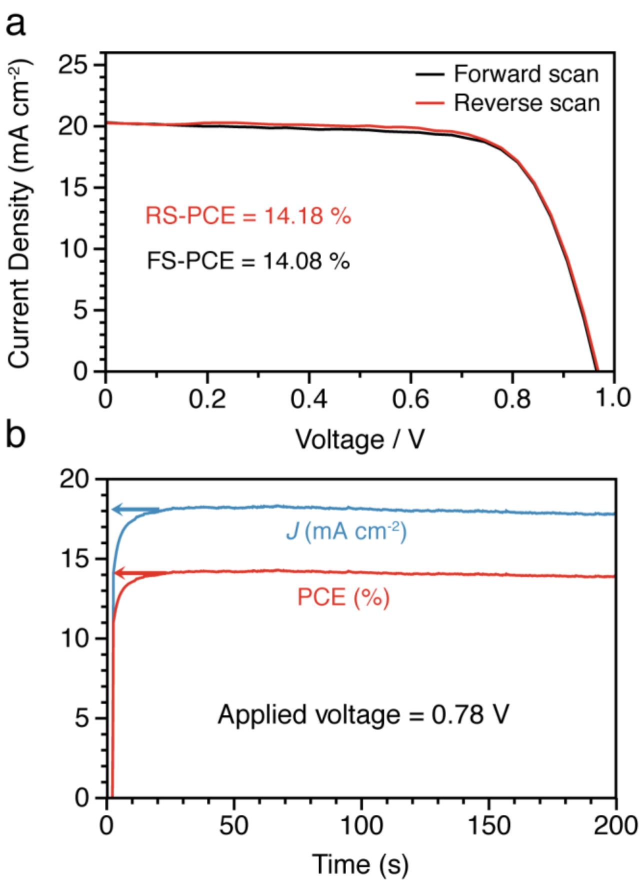

Supplementary Figure 7 | Stabilized power output characterization. (a) JV curves of a representative $CH_3NH_3PbI_{3-x}(SCN)_x$ based solar cell and (b) the corresponding stabilized power output in 200s.

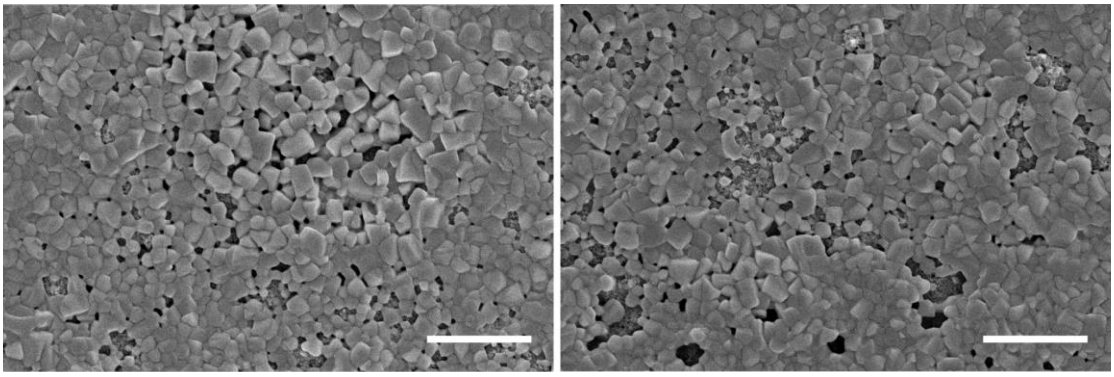

Supplementary Figure 8 | Morphology of perovskite films. SEM images of $CH_3NH_3PbI_3$ film prepared in ambient air with high humidity, the left and right images were obtained from two different films prepared at the same conditions. The scale bar is 2 μm.

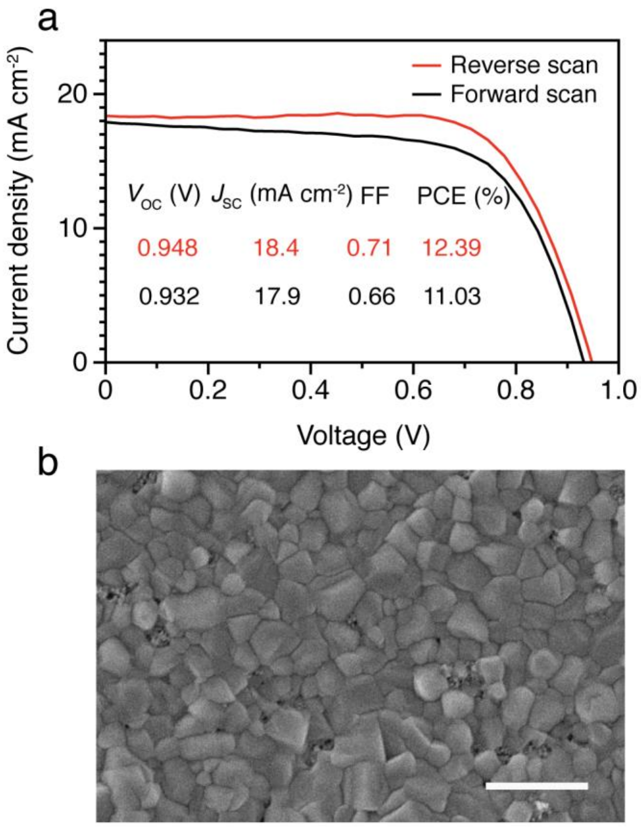

Supplementary Figure 9 | Morphology and photovoltaic performance of $CH_3NH_3PbI_3$ films prepared in a glovebox with PbI_2 precursor in DMSO solvent (a) J-V curves of $CH_3NH_3PbI_3$ based solar cell, (b) the corresponding SEM image of the $CH_3NH_3PbI_3$ film, the scale bar is 1μm.

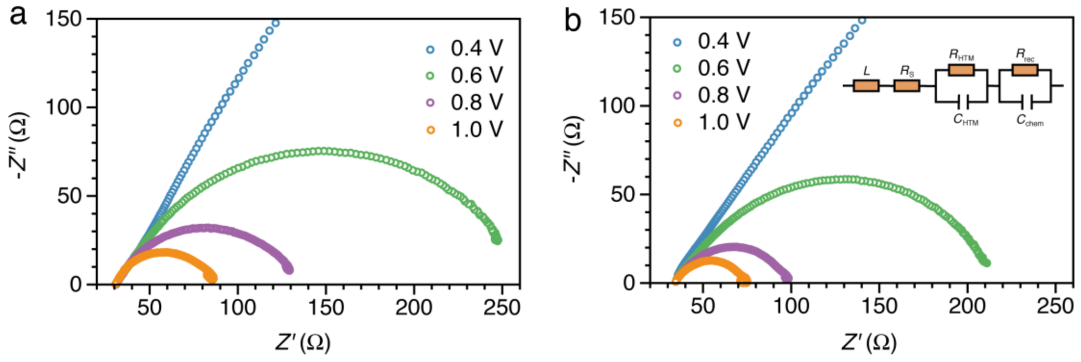

Supplementary Figure 10 | Impedance analysis. IS spectra of (a) $CH_3NH_3PbI_{3-x}(SCN)_x$ and (b) $CH_3NH_3PbI_3$ -based solar cells measured with different applied voltages in dark, the active area is 0.1 cm^2 for both devices. The inset in (b) is the equivalent circuit used for fitting the experimental data in the high and intermediate frequency regions and an additional inductive element (L) that accounts for the wire induction is added for a better fitting.

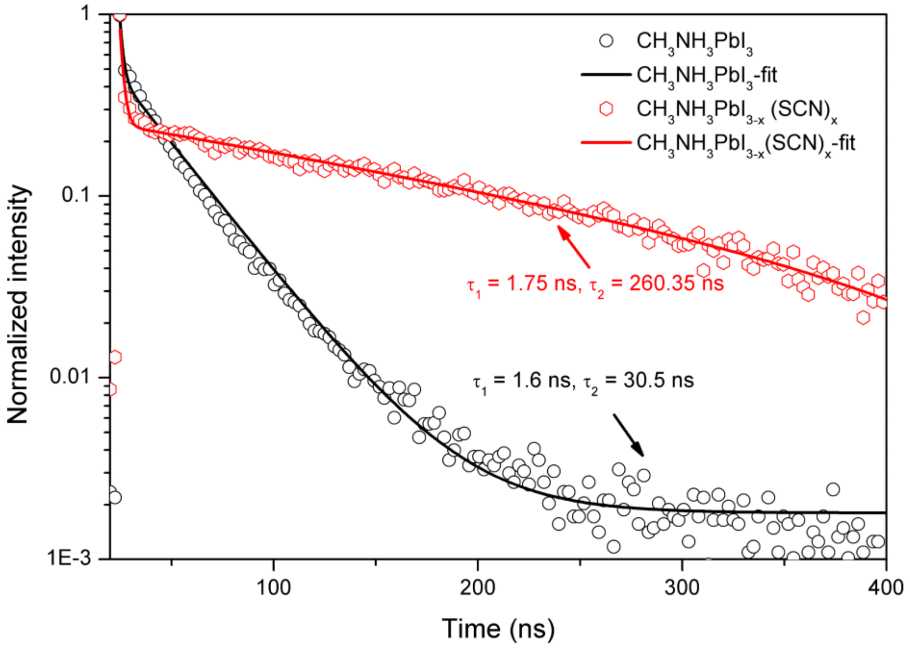

Supplementary Figure 11 | Time-resolved PL decay characterizations of $CH_3NH_3PbI_{3-x}(SCN)_x$ and $CH_3NH_3PbI_3$ films prepared in humid condition. The experimental data is fitted with two-component exponential decay.

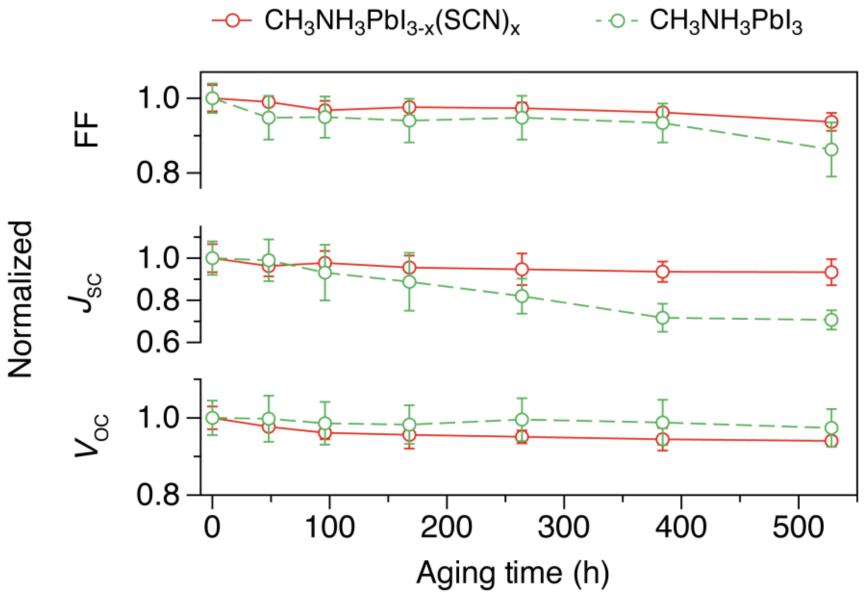

Supplementary Figure 12 | Long-term stability tests. Evolution of the open-circuit voltage (V_{OC}), short-circuit current density (J_{SC}), and fill factor (FF) of $CH_3NH_3PbI_{3-x}(SCN)_x$ and $CH_3NH_3PbI_3$ based solar cells during the aging test.

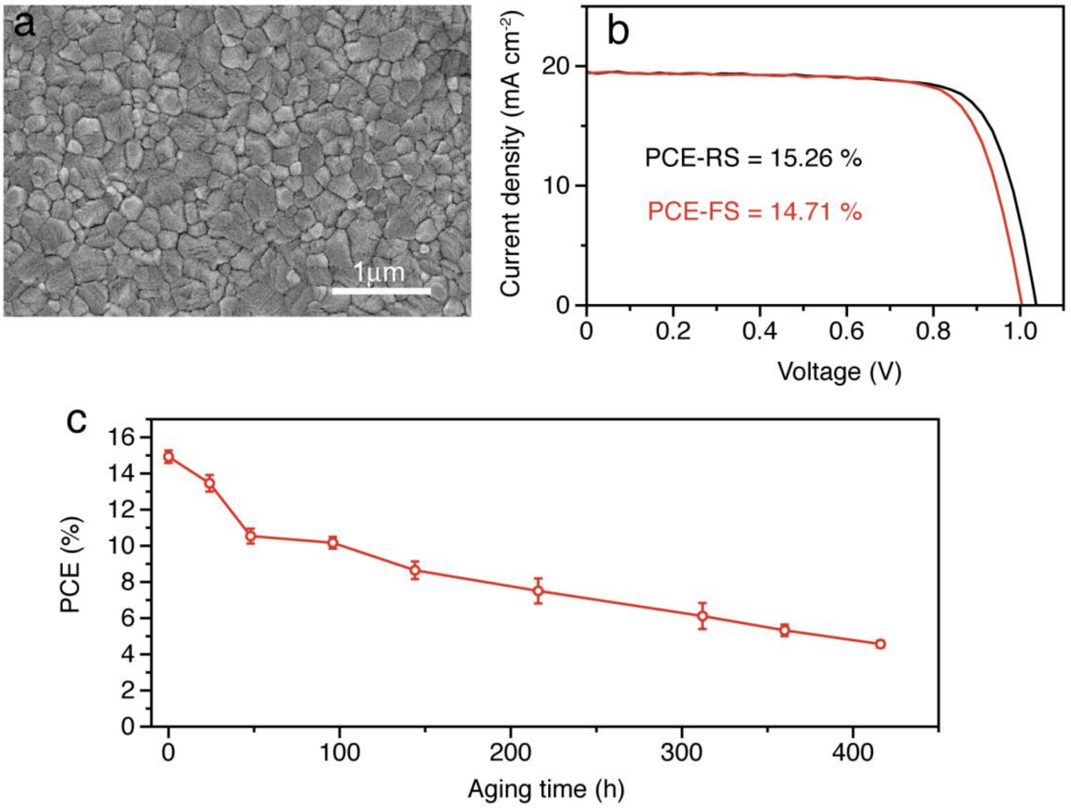

Supplementary Figure 13 | Morphology and photovoltaic performance of $CH_3NH_3PbI_3$ film prepared in a glovebox with PbI_2 precursor in DMF solvent (a) SEM image of the $CH_3NH_3PbI_3$ perovskite film, (b) *J-V* curves of the perovskite solar cells based on $CH_3NH_3PbI_3$, (c) Long-term stability of $CH_3NH_3PbI_3$ solar cells prepared in glovebox and characterized in air with the RH of ~80%.

(**Note**: *It is notable that the efficiency of the control device prepared in the glovebox degraded much faster than another control device prepared in ambient air. Because the former is prepared in pure N_2, it is reasonable to find that it is more sensitive to moisture than the latter prepared in humid air and thus has worse moisture tolerance.*)

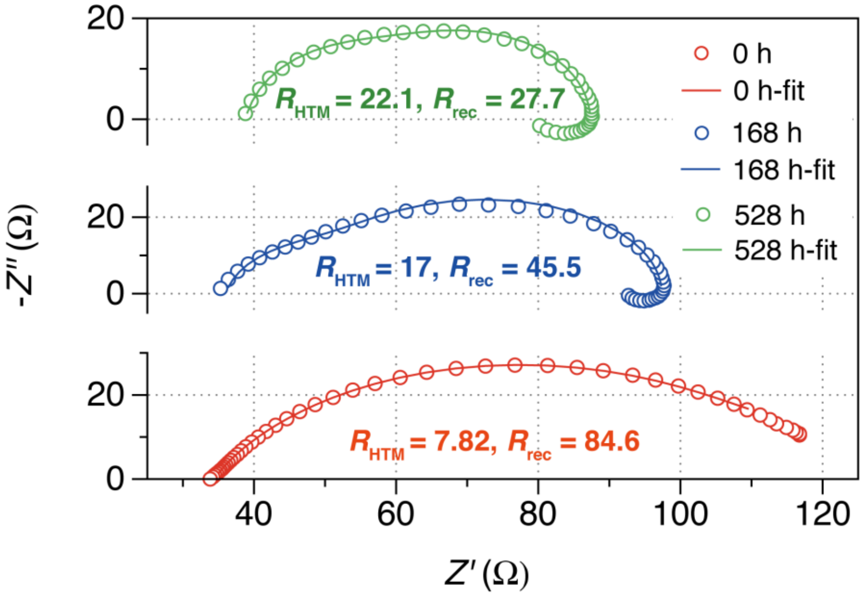

Supplementary Figure 14 | Impedance Analysis. IS spectra of $CH_3NH_3PbI_{3-x}(SCN)_x$ based solar cell with characteristic aging time of 0h, 168h, and 528h obtained in dark with a 0.9 V applied voltage.

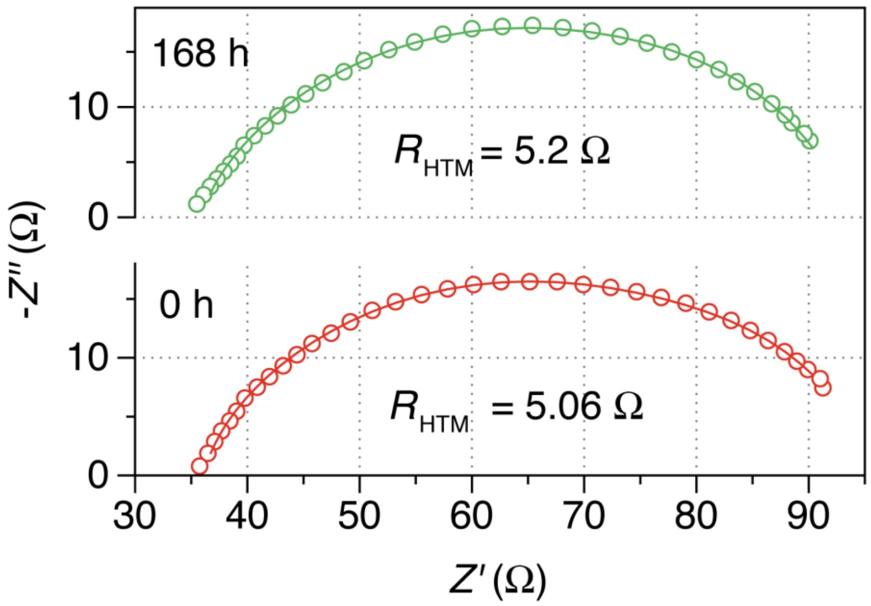

Supplementary Figure 15 | Impedance analysis. IS spectra of $CH_3NH_3PbI_{3-x}(SCN)_x$ based solar cell with air exposure time of 0h, 168h obtained in dark with a 1 V applied voltage, devices were kept in desiccator.

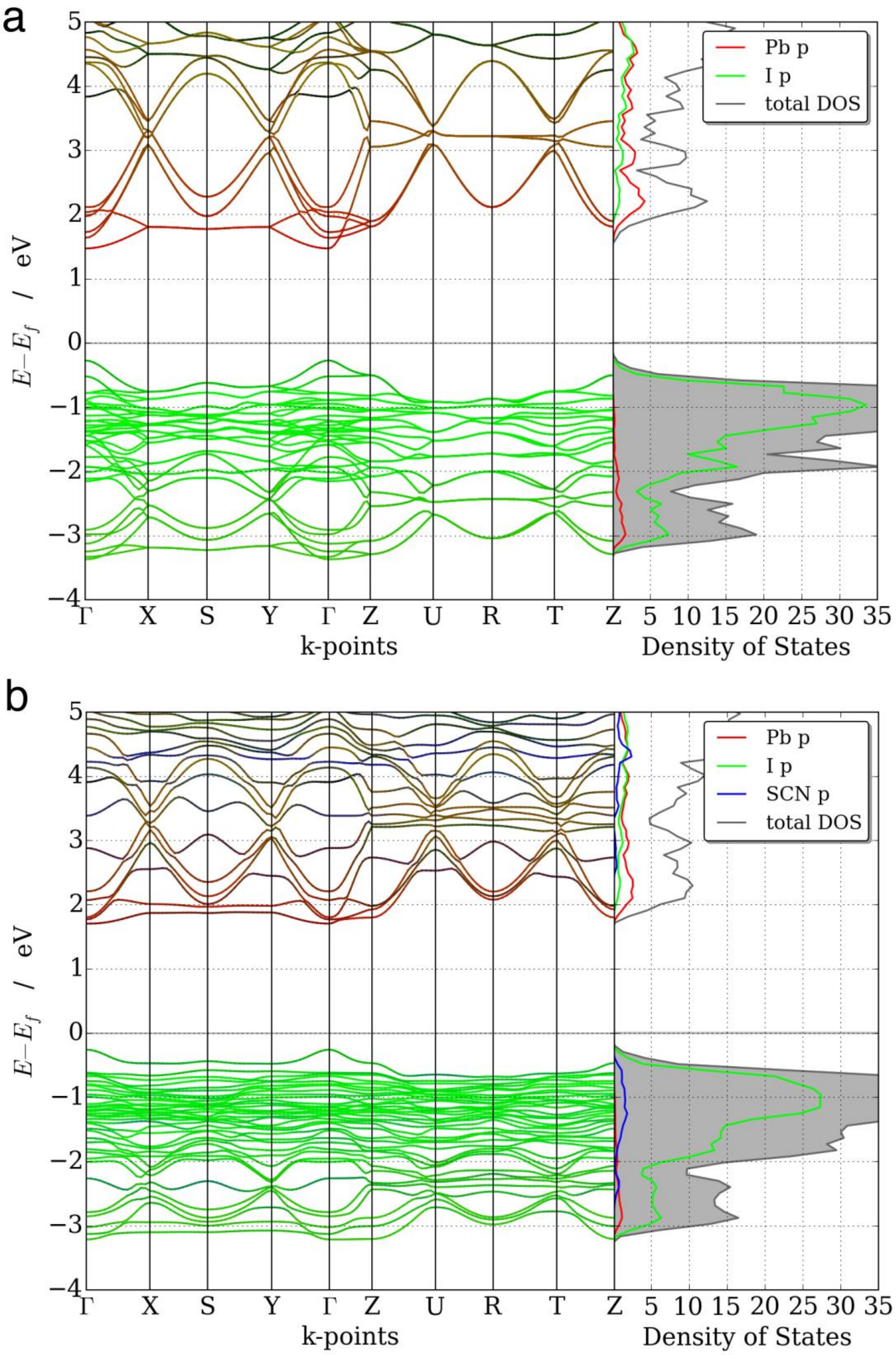

Supplementary Figure 16 | Band structure calculation. Calculated band structures of (a) $CH_3NH_3PbI_3$ and (b) $CH_3NH_3PbI_{3-x}(SCN)_x$ based on a chemical formula of $(CH_3NH_3)_4Pb_4I_{11}SCN$.

Supplementary Table 1 | Summary of cell parameters. Pholvoltaic performance of $CH_3NH_3PbI_{3-x}(SCN)_x$ and $CH_3NH_3PbI_3$ based solar cells measured under 100 mW cm^{-2} AM 1.5G illumination, the device area is $0.1 cm^2$.

RS/FS<br>Perovskite	V_{OC} (V)	J_{SC} (mA cm^{-2})	FF	PCE (%)
$CH_3NH_3PbI_{3-x}(SCN)_x$	0.956/0.963	21.1/20.9	0.75/0.72	15.12/14.52
$CH_3NH_3PbI_3$	0.905/0.898	15.3/14.9	0.634/0.599	8.78/8.02

Supplementary Table 2 | Summary of solar cell parameters during aging tests. PCE, R_{HTM}, and R_{rec} values of $CH_3NH_3PbI_{3-x}(SCN)_x$ obtained at different aging time.

Ageing time (h)	RS-/FS-PCE (%)	R_{HTM} (Ω)	R_{rec} (Ω)
0	12.02/11.83	7.9	62.2
168	10.79/9.26	15.5	32.4
528	9.97/7.45	21.9	22.7

Note: The R_{HTM} and R_{rec} values are derived from the according IS spectra measured with 1V applied voltage in dark, the device area is 0.1 cm^2.
